# Patients With Atopic Dermatitis Show Increased Clonal Hematopoiesis and Risk of Hematological Cancer

**DOI:** 10.1111/all.16587

**Published:** 2025-05-10

**Authors:** Verena Vogi, Emina Jukic, Robert Gruber, Andre Fiona, Deborah Minzaghi, Johannes Zschocke, Sandrine Dubrac

**Affiliations:** ^1^ Institute of Human Genetics Medical University of Innsbruck Innsbruck Austria; ^2^ Department of Dermatology, Venereology and Allergology Medical University of Innsbruck Innsbruck Austria

**Keywords:** atopic dermatitis, blood, cancer, clonal hematopoiesis of indeterminate potential, inflammation, skin

**Abbreviations**
AD: atopic dermatitis
CHIP: clonal hematopoiesis of indeterminate potential
CTRL: control cohort
FLG: filaggrin
VAF: variant allele frequency

To the Editor,

Atopic dermatitis (AD) is the most common chronic, inflammatory skin disease worldwide, characterized by recurrent eczematous skin lesions over dry skin and severe pruritus. AD is associated with several comorbidities, including other atopic disorders, skin infections, and malignancies. Epidemiological studies [1, 2], case reports, and studies in mouse models have demonstrated an association between AD and blood malignancies. The relative risk of lymphoma in AD patients increases with the frequency of hospitalization for patients with severe AD [3]. Clonal Hematopoiesis of Indeterminate Potential (CHIP) is defined as the presence of one or more somatic pathogenic variants (in genes commonly altered in hematological disorders) with > 2% variant allele frequency (VAF) in the peripheral blood of individuals without evidence of hematological malignancy, dysplasia, or unexplained cytopenias. Furthermore, CHIP is associated with a 0.5%–1.0% risk of progression to leukemia per year. In the current study, we sought early markers of hematological malignancies in AD patients. We screened 75 AD patients and 36 healthy individuals (Table S1) for CHIP (Tables S2 and S3), a condition that resembles a premalignant state of hematological diseases. CHIP was present in 20/75 (26.7%) of AD patients (Figure 1A), with a total of 22 variants in 10 different genes (Tables S4 and S5; Figure 1B). In contrast, a CHIP variant was present in only 1/36 (2.8%) of healthy individuals (Figure 1A). Taken together, these results indicate a 9.6‐fold increased probability for CHIP in AD patients compared to healthy individuals (*p* < 0.001, Figure 1A). VAFs ranged from 2% to 80% in AD patients (Figure 1C), suggesting a risk of malignant progression. Three AD patients (1F/2M aged 21, 28 and 30 years) displayed two CHIPs (Table S5 and Figure 1E). Two exhibited mild and one moderate AD symptoms, and they all received similar treatment, that is, topical cortisone and antihistamines as needed, and were never treated with systemic immunosuppressive drugs, biologics, or UV therapy. For all three, the onset of AD was in the first year of life. Additional analyses showed a higher risk of CHIP in patients treated with topical calcineurin inhibitors (Figure S1 and Table S6), demonstrating that systemic immunosuppressive therapy is not responsible for CHIP. Out of the 20 AD patients with CHIP, 12 were genotyped for *FLG* pathogenic variants. Data showed that only 3 AD patients with CHIP carried a *FLG* pathogenic variant: 1 with p.(Arg501*) and 2 with p.(Ser761fs*) (Figure 1E). However, 2 AD patients carrying a *FLG* pathogenic variant were exhibiting 2 CHIPs (Figure 1E). Moreover, the presence of CHIP was inversely associated with disease severity (*p* < 0.001) (Figure 1D) and independent of age, sex, and serum IgE and LDH levels (Table S6). In AD patients, most CHIP mutations (*KMT2C*, *KMT2D*, *PIK3CA*, *SOCS1*, *ATR*, *MEF2B*, and *RB1*) can be classified as lymphoid CHIP (L‐CHIP), in genes crucial for the differentiation and development of B‐cells, whereas only one CHIP‐mutation (EP300) involves a gene linked to myeloid cell differentiation, representing myeloid CHIP (M‐CHIP). Thus, these data show an increased probability for L‐CHIP in AD patients, in line with the literature associating AD with lymphoma [4]. Detailed analysis of genes with CHIP‐associated variants in AD patients showed that 36.4% of the variants in AD patients were located in *KMT2C* (Figure 2A and Table S4), while *KMT2D* was the second most frequently mutated gene (Figure 2A and Table S4). *SOCS1* is the third most frequently mutated gene (Table S4). Mutations in *KMT2C/D* and *SOCS1* are associated with blood malignancies because they interfere with the regulation of cell growth (e.g., epigenetic reprogramming and dysregulated gene expression or aberrant pathway activation). CHIP‐associated variants are located in different genes in AD patients and the healthy donor (Table S4), suggesting distinct mechanism(s) inducing mutagenesis in hematological cells in AD patients. CHIP in AD patients was found across all age groups (Figure 2B), with a mean age of 33 years, but the peak was situated between 24 and 26 years of age (Figure 2C). In young AD patients, CHIP mainly targets *KMT2C* (Table S7). In the general population, the prevalence of CHIP in peripheral blood is low from birth until the age of 50 (< 0.5%). Then, it increases and may affect 10–40% of people aged 60 and over, with a male predominance. CHIP mutations are also observed in patients aged 35 to 72 with autoimmune diseases such as rheumatoid arthritis (17%) [5]. In conclusion, these data provide the first insights into possible risk factors that increase the risk of developing hematological malignancies in patients with AD. Although this study has several limitations (e.g., small number of subjects, missing data on *FLG* genotyping and lack of data on gene copy number variations), it should encourage close follow‐up of AD patients and further studies in order to develop preventive interventions.

**FIGURE 1:**
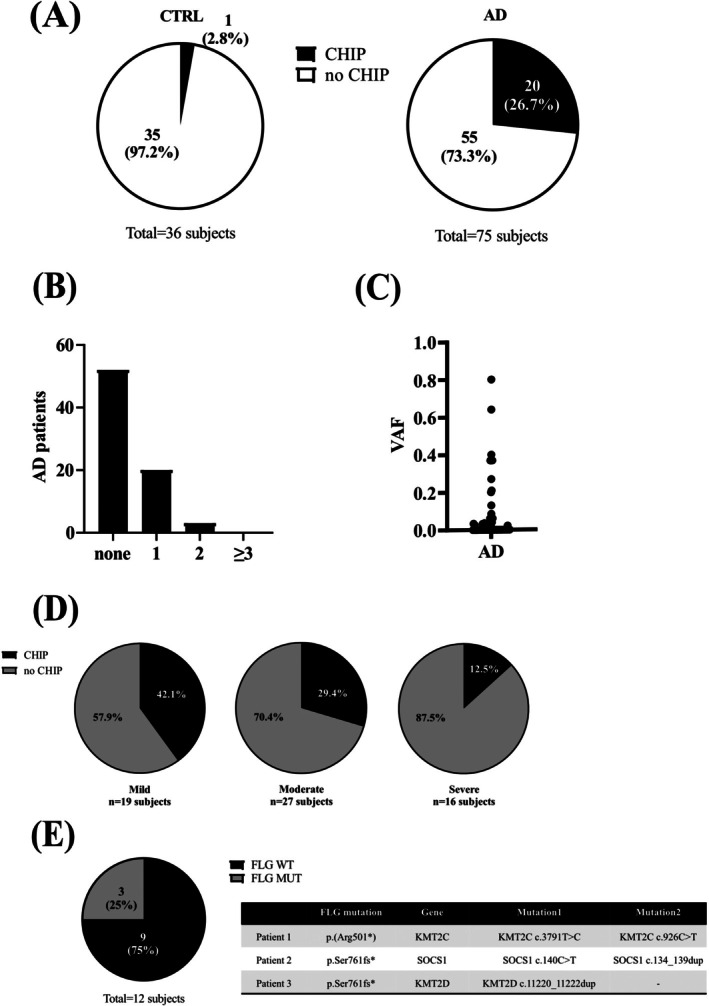
Increased CHIP and VAF in patients with atopic dermatitis. (A) Percentages of atopic dermatitis (AD) patients and healthy individuals (CTRL) with Clonal Hematopoiesis of Indeterminate Potential (CHIP) pathogenic variant, (B) Numbers of CHIP pathogenic variants per patient with AD and (C) Variant Allele Frequency (VAF) distribution of CHIP pathogenic variants in AD patients. (D) Percentages of AD patients with CHIP according to disease severity. (E) Percentages of AD patients with CHIP according to the presence or absence of FLG pathogenic variants and table showing the CHIP mutations in the three AD patients with FLG pathogenic variants. Data were analyzed with a chi‐square test or a Student's *t*‐test.

**FIGURE 2:**
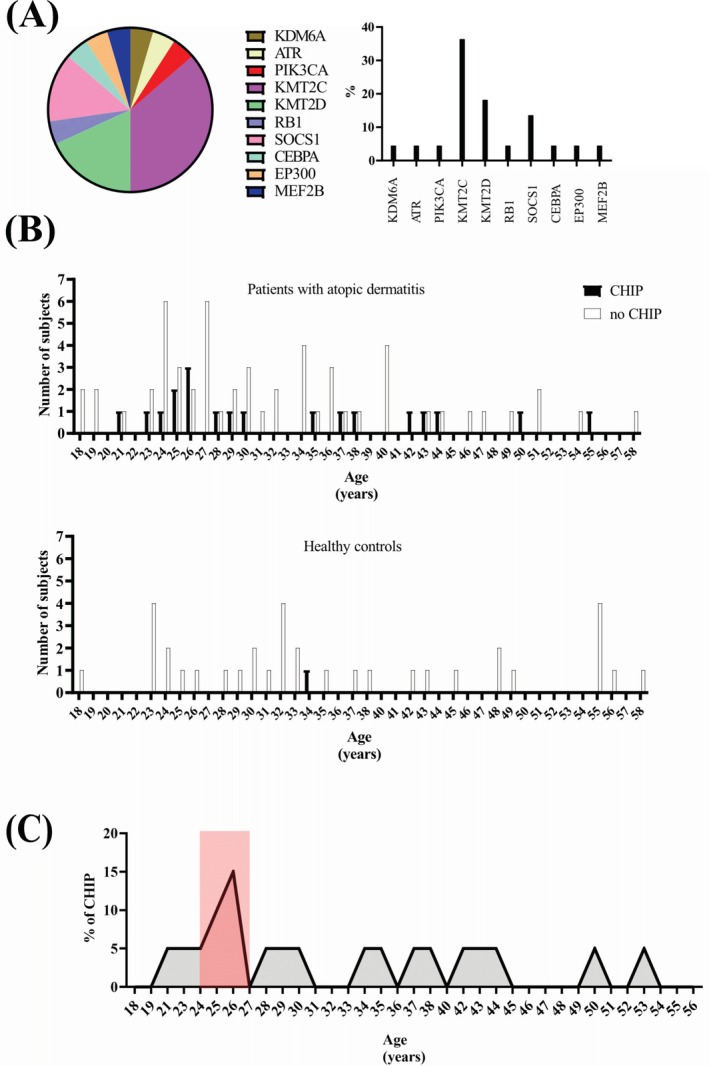
Genes affected by CHIP mutations in patients with atopic dermatitis and effects of FLG null mutations: (A) Percentages of genes affected by Clonal Hematopoiesis of Indeterminate Potential (CHIP) pathogenic variants in patients with atopic dermatitis (AD). (B) Numbers of subjects displaying or not displaying a CHIP according to age. (C) Percentages of CHIP according to AD patients' age. Data were analyzed with a chi‐square test.

## Author Contributions

V.V.: data acquisition, analysis and curation, manuscript writing; E.J., J.Z.: methodology and conceptualization, manuscript revision, funding acquisition; R.G., D.M., and A.F.: subjects' recruitment, manuscript revision; S.D.: conceptualization and supervision of the project, data retrieval, analysis and curation, manuscript writing, and funding acquisition. All authors approved the manuscript.

## Conflicts of Interest

The authors declare no conflicts of interest.

## Supporting information


Figure S1.

Table S1.
Table S2.
Table S3.
Table S4.
Table S5.
Table S6.
Table S7.

## Data Availability

The data that supports the findings of this study are available in the supplementary material of this article.
