# Supplementary material for: Patients With Atopic Dermatitis Show Increased Clonal Hematopoiesis and Risk of Hematological Cancer
Source: Allergy. 2025 May 10;80(9):2646–9. doi: 10.1111/all.16587 (PMC12444847; doi:10.1111/all.16587)
Supplement: Supplementary file 1 — Figure S1. [file ALL-80-2646-s002.docx]

**Supplementary information**

**METHODS**

A total of 111 samples were screened, including 75 AD patients and 36 healthy individuals. Age below 18 or above 60 years as well as the presence of WHO-defined hematological malignancy, dysplasia, or unexplained cytopenias were exclusion criteria for all subjects. Inflammatory skin disease or allergies were additional exclusion criteria for healthy individuals. The study was approved by the Ethics Committee of the Medical University of Innsbruck and conducted in accordance with the tenets of the Declaration of Helsinki. All study subjects gave written informed consent and participated voluntarily [(AN2016-0260 368/4.22 453/AM1 (4577a), UN5073 325/4.2, UN4253 297/4.48]. A number of patients (n=34) and healthy individuals (n=16) were screened for *FLG* pathogenic variants by exome sequencing or as previously described^(1)^. Study cohort characteristics are summarized in Table S1.

**2.2 Sample Preparation**

Genomic DNA was obtained from fresh peripheral blood samples using the DNeasy Blood & Tissue Kit (Qiagen, Hilden, Germany) according to the manufacturer’s instructions. DNA concentration and quality were assessed via Nanodrop photometry and via Qubit dsDNA BR Assay Kit on a Qubit Fluorometer (Thermo Fisher Scientific, Waltham, USA).

**2.3 Massive Parallel Sequencing**

For the identification of CHIP-associated variants, DNA samples were analyzed by massively-parallel sequencing using a hybridization-capture enrichment-based sequencing approach (Illumina, San Diego, USA). Library quality, fragment size and library quantity were assessed via the HighSensitiy DNA Chip for BioAnalyzer (Agilent, Santa Clara, USA). The gene panel for enrichment is of custom design and covers a range of 153 genes associated with malignancies, 97 of which are predominantly involved in myeloid and lymphoid neoplasms and their precursors. Only genes associated at least once with CHIP or a hematologic neoplasm according to previous studies were included in the analysis. An overview of the genes analyzed is provided in Table S2. Sequencing was performed on a NextSeq instrument (Illumina, San Diego, USA) according to the manufacturer’s instructions.

**2.4 Variant Calling and Annotation Strategies**

For data analysis and variant calling, the SeqNext Module from JSI Medical Systems (Ettenheim, DE) was utilized to align raw sequence reads to the hg19 build of the human reference genome. Regions with a minimum coverage of 50 were analyzed. Common single nucleotide polymorphisms with a minor allele frequency of at least 1% in the Non-Finnish European population and synonymous changes were excluded. In addition, variants present in more than 10 % of the cohort and in more than 10% of patients per sequencing run, and variants in areas of excessive local noise, were considered technical artefacts and excluded from the dataset. Only coding variants and variants -2/+2 of the coding region with a VAF of at least 2%, a quality score of 30 or higher and a variant read count of at least 15 were included. In addition, variants with a VAF of 0.40 to 0.60 and 0.85 to 1.00 were excluded from the results to exclude potential germline variants. Among the remaining variants, likely benign variants were identified using MLLi:db (Database of Münchner Leukämie Labor, https://mlli.com), Cosmic (Catalogue of Somatic Mutations in Cancer, https://cancer.sanger.ac.uk/cosmic), HGMD (Human Gene Mutation Database, https://ihgseq13.helmholtz-muenchen.de/hgmd/pro/search_gene.php), ClinVar (https://www.ncbi.nlm.nih.gov/clinvar) and LOVD (Leiden Open Variation Database, https://www.lovd.nl/).

**2.5 Statistical Analysis**

Statistical Analysis was performed using SPSS (IBM Corp. Released 2019. IBM SPSS Statistics for Windows, Version 26.0. Armonk, NY: IBM Corp) or GraphPad prism 9.4.1. To explore differences in traits between AD patients and controls, a two-tailed Student’s t-test was performed. Alternatively, a Chi-Square-Test was carried out. Statistical significance was defined as p < 0.05.

**Reference**

1. Blunder S, Rühl R, Moosbrugger-Martinz V, Krimmel C, Geisler A, Zhu H, et al. Alterations in Epidermal Eicosanoid Metabolism Contribute to Inflammation and Impaired Late Differentiation in FLG-Mutated Atopic Dermatitis. J Invest Dermatol. 2017;137(3):706-15.
