# Supplementary material for: Patients With Atopic Dermatitis Show Increased Clonal Hematopoiesis and Risk of Hematological Cancer
Source: Allergy. 2025 May 10;80(9):2646–9. doi: 10.1111/all.16587 (PMC12444847; doi:10.1111/all.16587)
Supplement: Supplementary file 2 — Table S1. Table S2. Table S3. Table S4. Table S5. Table S6. Table S7. [file ALL-80-2646-s001.docx]

**Table S1: Description of cohorts**

|  | **CTRL** | **AD** | **P value** |
| --- | --- | --- | --- |
| Number | 36 | 75 |  |
| Age | 36.3±2.0 (36) | 32.4±1.1 (75) | p=0.066 |
| Sex | 21F/15M (36) | 46F/29M (75) |  |
| *FLG* mutation  p.(Arg501*)  p.Ser761fs*  p.(Glu32*) | 0/16 | 13/33  6/34  6/34  1/34 |  |
| Disease severity  Mild  Moderate  Severe |  | 20/45  27/45  15/45 |  |
| IgE | 33.5±16.6 (12) | 1863.0±513.4 (41) | p=0.046 |
| LDH | 158.3±6.8 (12) | 174.6±12.5 (21) | p=0.3579 |

Student’s t test for continuous variables, number of analyzed individual into brackets.

AD: atopic dermatitis; CTRL: healthy individuals, F: female, M: male.

**Table S2: Overview of analyzed genes**

|  | Chromosomes | Genes | CHIP-category | Exons | NM-Nr. |
| --- | --- | --- | --- | --- | --- |
| 1 | X | *BCOR* | M-CHIP | Full | NM_001123385.2 |
| 2 | X | *BCORL1* | M-CHIP | Full | NM_021946.4 |
| 3 | X | *KDM6A* | M-CHIP | Full | NM_001291415.2 |
| 4 | X | *PHF6* | M-CHIP | Full | NM_001015877.1 |
| 5 | X | *STAG2* | M-CHIP | Full | NM_001042750.2 |
| 6 | X | *ZRSR2* | M-CHIP | Full | NM_005089.4 |
| 7 | 1 | *CSF3R* | M-CHIP | 14-17 | NM_156039.3 |
| 8 | 1 | *MPL* | M-CHIP | Full | NM_005373.3 |
| 9 | 1 | *NRAS* | M-CHIP | 2-4 | NM_002524.4 |
| 10 | 2 | *DNMT3A* | M-CHIP | Full | NM_022552.5 |
| 11 | 2 | *IDH1* | M-CHIP | 4 | NM_005896.3 |
| 12 | 2 | *SF3B1* | M-CHIP | 13-16 | NM_012433.3 |
| 13 | 3 | *GATA2* | M-CHIP | Full | NM_032638.5 |
| 14 | 4 | *KIT* | M-CHIP | Full | NM_000222.3 |
| 15 | 4 | *TET2* | M-CHIP | Full | NM_001127208.3 |
| 16 | 5 | *NPM1* | M-CHIP | 11 | NM_002520.6 |
| 17 | 7 | *BRAF* | M-CHIP | 8-? | NM_004333.5 |
| 18 | 7 | *CUX1* | M-CHIP | Full | NM_001202543 |
| 19 | 7 | *EZH2* | M-CHIP | Full | NM_004456.4 |
| 20 | 7 | *IKZF1* | M-CHIP | Full | NM_006060.6 |
| 21 | 8 | *MYC* | M-CHIP | Full | NM_002467.6 |
| 22 | 8 | *RAD21* | M-CHIP | Full | NM_006265.3 |
| 23 | 9 | *CDKN2A* | M-CHIP | Full | NM_000077.5 |
| 24 | 9 | *JAK2* | M-CHIP | Full | NM_004972.4 |
| 25 | 10 | *SMC3* | M-CHIP | Full | NM_005445.4 |
| 26 | 11 | *CBL* | M-CHIP | 8-9 | NM_005188.3 |
| 27 | 11 | *WT1* | M-CHIP | 7-9 | NM_024426.2 |
| 28 | 12 | *ETNK1* | M-CHIP | 3 | NM_018638.4 |
| 29 | 12 | *ETV6* | M-CHIP | Full | NM_001987.5 |
| 30 | 12 | *KRAS* | M-CHIP | 2-4 | NM_033360.3 |
| 31 | 12 | *PTPN11* | M-CHIP | Full | NM_002834.5 |
| 32 | 12 | *SH2B3* | M-CHIP | Full | NM_005475.3 |
| 33 | 13 | *FLT3* | M-CHIP | 14, 15, 20 | NM_004119.2 |
| 34 | 15 | *IDH2* | M-CHIP | 4 | NM_002168.3 |
| 35 | 16 | *CREBBP* | M-CHIP | Full | NM_004380.3 |
| 36 | 17 | *NF1* | M-CHIP | Full | NM_001042492.3 |
| 37 | 17 | *PPM1D* | M-CHIP | Full | NM_003620.4 |
| 38 | 17 | *SRSF2* | M-CHIP | 1 | NM_003016.4 |
| 39 | 17 | *TP53* | M-CHIP | Full | NM_000546.6 |
| 40 | 18 | *SETBP1* | M-CHIP | 4 | NM_015559.2 |
| 41 | 19 | *CALR* | M-CHIP | 9 | NM_004343.3 |
| 42 | 19 | *CEBPA* | M-CHIP | Full | NM_004364.5 |
| 43 | 20 | *ASXL1* | M-CHIP | 13 | NM_015338.5 |
| 44 | 20 | *GNAS* | M-CHIP | 8-9 | NM_004119.2 |
| 45 | 21 | *RUNX1* | M-CHIP | Full | NM_001754.5 |
| 46 | 21 | *U2AF1* | M-CHIP | 2, 6 | NM_006758.2 |
| 47 | 22 | *EP300* | M-CHIP | Full | NM_001429.4 |
| 48 | X | *BTK* | L-CHIP | 11, 15, 16 | NM_000061.2 |
| 49 | X | *DDX3X* | L-CHIP | Full | NM_001356.5 |
| 50 | 1 | *ARID1A* | L-CHIP | Full | NM_006015.6 |
| 51 | 1 | *FAM46C* | L-CHIP | Full | NM_017709.4 |
| 52 | 1 | *ID3* | L-CHIP | Full | NM_002167.5 |
| 53 | 1 | *JAK1* | L-CHIP | 16, 20-24 | NM_002227.4 |
| 54 | 1 | *NOTCH2* | L-CHIP | 26-28, 34 | NM_024408.4 |
| 55 | 1 | *SPEN* | L-CHIP | Full | NM_015001.3 |
| 56 | 1 | *TNFRSF14* | L-CHIP | Full | NM_003820.4 |
| 57 | 2 | *ALK* | L-CHIP | Full | NM_04304 |
| 58 | 2 | *CXCR4* | L-CHIP | Full | NM_003467.3 |
| 59 | 2 | *XPO1* | L-CHIP | 15-16 | NM_003400.3 |
| 60 | 3 | *ATR* | L-CHIP | Full | NM_001184.4 |
| 61 | 3 | *KLHL6* | L-CHIP | Full | NM_130446.4 |
| 62 | 3 | *MYD88* | L-CHIP | Full | NM_002468.5 |
| 63 | 3 | *PIK3CA* | L-CHIP | Full | NM_006218.4 |
| 64 | 3 | *RHOA* | L-CHIP | 2 | NM_001664.4 |
| 65 | 4 | *FBXW7* | L-CHIP | 8-12 | NM_033632.3 |
| 66 | 4 | *MAP2K1* | L-CHIP | Full | NM_002755.4 |
| 67 | 6 | *HIST1H1B* | L-CHIP | Full | NM_005322.3 |
| 68 | 6 | *NFKBIE* | L-CHIP | Full | NM_004556.3 |
| 69 | 6 | *SGK1* | L-CHIP | Full | NM_001143676.3 |
| 70 | 6 | *TNFAIP3* | L-CHIP | Full | NM_001270508.2 |
| 71 | 7 | *CARD11* | L-CHIP | Full | NM_032415.7 |
| 72 | 7 | *KMT2C* | L-CHIP | Full | NM_170606.2 |
| 73 | 7 | *POT1* | L-CHIP | Full | NM_015450.3 |
| 74 | 8 | *UBR5* | L-CHIP | 58 | NM_015902.5 |
| 75 | 9 | *ABL1* | L-CHIP | 4-9 | NM_005157.5 |
| 76 | 9 | *NOTCH1* | L-CHIP | 26-28, 34 | NM_017617.5 |
| 77 | 9 | *PAX5* | L-CHIP | Full | NM_016734.3 |
| 78 | 9 | *PTPRD* | L-CHIP | Full | NM_002839.4 |
| 79 | 10 | *PTEN* | L-CHIP | Full | NM_000314.8 |
| 80 | 10 | *RET* | L-CHIP | 16 | NM_020975 |
| 81 | 11 | *ATM* | L-CHIP | Full | NM_000051 |
| 82 | 11 | *BIRC3* | L-CHIP | Full | NM_001165.5 |
| 83 | 11 | *CCND1* | L-CHIP | Full | NM_053056.3 |
| 84 | 12 | *CCND3* | L-CHIP | Full | NM_001760.5 |
| 85 | 12 | *KMT2D* | L-CHIP | Full | NM_003482.4 |
| 86 | 13 | *DIS3* | L-CHIP | Full | NM_014953.5 |
| 87 | 13 | *FOXO1* | L-CHIP | Full | NM_002015.4 |
| 88 | 13 | *RB1* | L-CHIP | Full | NM_000321.3 |
| 89 | 14 | *TRAF3* | L-CHIP | Full | NM_145725.3 |
| 90 | 15 | *B2M* | L-CHIP | Full | NM_004048.4 |
| 91 | 16 | *PLCG2* | L-CHIP | Full | NM_002661.5 |
| 92 | 16 | *SOCS1* | L-CHIP | Full | NM_003745.2 |
| 93 | 17 | *BRCA1* | L-CHIP | Full | NM_007294.4 |
| 94 | 17 | *CD79B* | L-CHIP | Full | NM_000626.4 |
| 95 | 17 | *GNA13* | L-CHIP | Full | NM_006572.6 |
| 96 | 17 | *STAT3* | L-CHIP | 20-21 | NM_139276.2 |
| 97 | 17 | *STAT5B* | L-CHIP | 16 | NM_012448.3 |
| 98 | 18 | *BCL2* | L-CHIP | Full | NM_000633.3 |
| 99 | 19 | *JAK3* | L-CHIP | Full | NM_000215.4 |
| 100 | 19 | *KLF2* | L-CHIP | Full | NM_016270.4 |
| 101 | 19 | *MEF2B* | L-CHIP | Full | NM_001145785.2 |
| 102 | 19 | *TCF3* | L-CHIP | 18 | NM_001136139.4 |
| 103 | 22 | *CHEK2* | L-CHIP | Full | NM_007194.4 |

**Table S3: List of identified CHIP-associated variants**

| Chromosome | Gene | Exon | Locus | Variant (cDNA) | Variant (Protein) | VAF (%) | Variant Reads | Total Coverage |
| --- | --- | --- | --- | --- | --- | --- | --- | --- |
| X | *KDM6A* | E23 | X:44942715 | c.3295C>T | p.(Gln1099Ter) | 21.00 | 149 | 710 |
| 3 | *ATR* | E10 | 3:142555898 | c.2320del | p.(Ile774TyrfsTer5) | 5.20 | 145 | 2788 |
| 3 | *PIK3CA* | E13 | 3:179219967 | c.1930T>C | p.(Tyr644His) | 3.40 | 39 | 1147 |
| 3 | *PIK3CA* | E13 | 3:179219967 | c.1930T>C | p.(Tyr644His) | 5.30 | 89 | 1679 |
| 7 | *KMT2C* | E7 | 7:152273860 | c.857C>A | p.(Ala286Glu) | 4.00 | 195 | 4875 |
| 7 | *KMT2C* | E7 | 7:152273929 | c.926C>T | p.(Pro309Leu) | 6.10 | 386 | 6328 |
| 7 | *KMT2C* | I7 | 7:152265211 | c.1013-2A>G | p.? | 3.60 | 46 | 1277 |
| 7 | *KMT2C* | E8 | 7:152265168 | c.1054G>A | p.(Asp352Asn) | 6.10 | 138 | 2262 |
| 7 | *KMT2C* | E18 | 7:152266990 | c.2876T>C | p.(Met959Thr) | 3.20 | 223 | 6969 |
| 7 | *KMT2C* | E20 | 7:152224170 | c.3168G>A | p.(Trp1056Ter) | 2.00 | 44 | 2200 |
| 7 | *KMT2C* | E24 | 7:152207350 | c.3791T>C | p.(Val1264Ala) | 2.50 | 26 | 1040 |
| 7 | *KMT2C* | E28 | 7:152207932-152207933 | c.4373_4374delinsCC | p.(His1458Pro) | 37.00 | 679 | 1835 |
| 12 | *KMT2D* | E39 | 12:49033482-49033483 | c.11220_11222dup | p.(Gln3745dup) | 37.00 | 264 | 714 |
| 12 | *KMT2D* | E39 | 12:49032988-49032989 | c.11714_11716dup | p.(Gln3905dup) | 37.00 | 844 | 2281 |
| 12 | *KMT2D* | E39 | 12:49032840 | c.11853G>T | p.(Gln3951His) | 2.20 | 34 | 1545 |
| 12 | *KMT2D* | E39 | 12:49032842-49032860 | c.11855_11863del | p.(Gln3952_Gln3954del) | 37.00 | 798 | 2157 |
| 13 | *RB1* | E9 | 13:48364929 | c.897T>A | p.(Phe299Leu) | 40.00 | 291 | 728 |
| 16 | *CREBBP* | E31 | 16:3728302-3728304 | c.6743_6745del | p.(Gln2248del) | 2.20 | 25 | 1136 |
| 16 | *SOCS1* | E2 | 16:11255336-11255378 | c.108_143del | p.(Arg38_Ala49del) | 80.00 | 1003 | 1254 |
| 16 | *SOCS1* | E2 | 16:11255339-11255340 | c.134_139dup | p.(Val45_Pro46dup) | 13.00 | 88 | 676 |
| 16 | *SOCS1* | E2 | 16:11255339 | c.140C>T | p.(Ala47Val) | 20.00 | 122 | 610 |
| 19 | *CEBPA* | E1 | 19:33301849 | c.566C>A | p.(Pro189His) | 8.50 | 10 | 118 |
| 19 | *MEF2B* | E13 | 19:19145985 | c.919C>A | p.(Arg307Ser) | 27.00 | 100 | 370 |
| 22 | *EP300* | E14 | 22:41149885 | c.2504T>C | p.(Val835Ala) | 64.00 | 81 | 127 |

VAF: variant allele frequency

**Table S4: List of identified CHIP-associated pathogenic variants.**

| Study group | Chromosome | Gene | Exon | Genomic Locus (GRCh38) | Variant (cDNA) |
| --- | --- | --- | --- | --- | --- |
| CTRL | 16 | *CREBBP* | E31 | 16:3728302-3728304 | *CREBBP* c.6743_6745del |
| AD | X | *KDM6A* | E23 | X:44942715 | *KDM6A* c.3295C>T |
| AD | 3 | *ATR*  *PIK3CA* | E10  E13 | 3:142555898 3:179219967 | *ATR* c.2320del  *PIK3CA* c.1930T>C |
| AD | 7 | *KMT2C* | E7  E7  I7  E8  E18  E20  E24  E28 | 7:152273860  7:152273929  7:152265211  7:152265168  7:152266990  7:152224170  7:152207350 7:152207932-152207933 | *KMT2C* c.857C>A  *KMT2C* c.926C>T  *KMT2C* c.1013-2A>G  *KMT2C* c.1054G>A  *KMT2C* c.2876T>C  *KMT2C* c.3168G>A  *KMT2C* c.3791T>C  *KMT2C* c.4373_4374delinsCC |
| AD | 12 | *KMT2D* | E39  E39  E39  E39 | 12:49033482-49033483  12:49032988-49032989  12:49032840  12:49032842-49032860 | *KMT2D* c.11220_11222dup  *KMT2D* c.11714_11716dup  *KMT2D* c.11853G>T  *KMT2D* c.11855_11863del |
| AD | 13 | *RB1* | E9 | 13:48364929 | *RB1* c.897T>A |
| AD | 16 | *SOCS1* | E2  E2  E2 | 16:11255336-11255378  16:11255339-11255340  16:11255339 | *SOCS1* c.108_143del  *SOCS1* c.134_139dup  *SOCS1* c.140C>T |
| AD | 19 | *CEBPA*  *MEF2B* | E1  E13 | 19:33301849  19:19145985 | *CEBPA* c.566C>A  *MEF2B* c.919C>A |
| AD | 22 | *EP300* | E14 | 22:41149885 | *EP300* c.2504T>C |

CTRL: healthy individual: AD: atopic dermatitis

**Table S5: List of pathogenic variants in patients with atopic dermatitis**

| **Patient Nr.** | **Mutation 1** | **Mutation 2** |
| --- | --- | --- |
| 1 | PIK3CA c.1930T>C |  |
| 2 | MEF2B c.919C>A |  |
| 3 | KMT2C c.3791T>C | KMT2C c.926C>T |
| 4 | KMT2D c.11853G>T |  |
| 5 | KMT2C c.1013-2A>G | KMT2D c.11855_11863del |
| 6 | KMT2C c.857C>A |  |
| 7 | PIK3CA c.1930T>C |  |
| 8 | KMT2C c.3168G>A |  |
| 9 | SOCS1 c.108_143del |  |
| 10 | RB1 c.897T>A |  |
| 11 | SOCS1 c.140C>T | SOCS1 c.134_139dup |
| 12 | KMT2D c.11220_11222dup |  |
| 13 | KMT2C c.1054G>A |  |
| 14 | EP300 c.2504T>C |  |
| 15 | KMT2C c.4373_4374delinsCC |  |
| 16 | ATR c.2320del |  |
| 17 | KMT2D c.11714_11716dup |  |
| 18 | KMT2C c.2876T>C |  |
| 19 | CEBPA c.566C>A |  |
| 20 | KDM6A c.3295C>T |  |

**Table S6: Cross-relationship between parameters**

| **Test Khi-deux de Pearson** | **P values** |
| --- | --- |
| Disease*Chromosome | p=0.344 |
| Disease**FLG* mutation | p=0.002 |
| Disease*Age | p=0.163 |
| Disease*Gene | p=0.013 |
| Disease*Sex | p=0.995 |
| CHIP**FLG* mutation | p=0.791 |
| CHIP*Age | p=0.787 |
| CHIP*Disease severity | p<0.001 |
| CHIP*Sex | p=0.960 |
| CHIP*Serum IgE | p=0.508 |
| CHIP*Serum LDH | p=0.236 |
| Gene*Sex | p=0.457 |
| Gene**FLG* mutation | p=0.786 |
| Gene*Age | p=0.151 |
| CHIP*Treatment | p<0.001 |
| CHIP*Topical cortisone | p=0.068 |
| CHIP*Topical calcineurin inhibitors | p=0.020 |
| CHIP*Systemic immunosuppressors | p=0.990 |
| CHIP*UV Therapy | p=870 |
| CHIP*Biologics | p=0.655 |
| CHIP*Antihistaminic | p=0.0117 |

Chi-Square test for categorical variables

**Table S7: Genes exhibiting a CHIP according to AD patients’ age**

| **Age (y)** | **Gene** |
| --- | --- |
| 21 | KMT2C |
| 23 | PIK3CA |
| 24 | KMT2C |
| 25 | KMT2D |
| 26 | MEF2B, KMT2C, KDM6A |
| 28 | KMT2C |
| 29 | CEBPA |
| 30 | SOCS1 |
| 35 | PIK3CA |
| 37 | ATR |
| 38 | KMT2D |
| 42 | RB1 |
| 43 | KMT2D |
| 44 | SOCS1 |
| 50 | KMT2C |
| 55 | KMT2C |

**
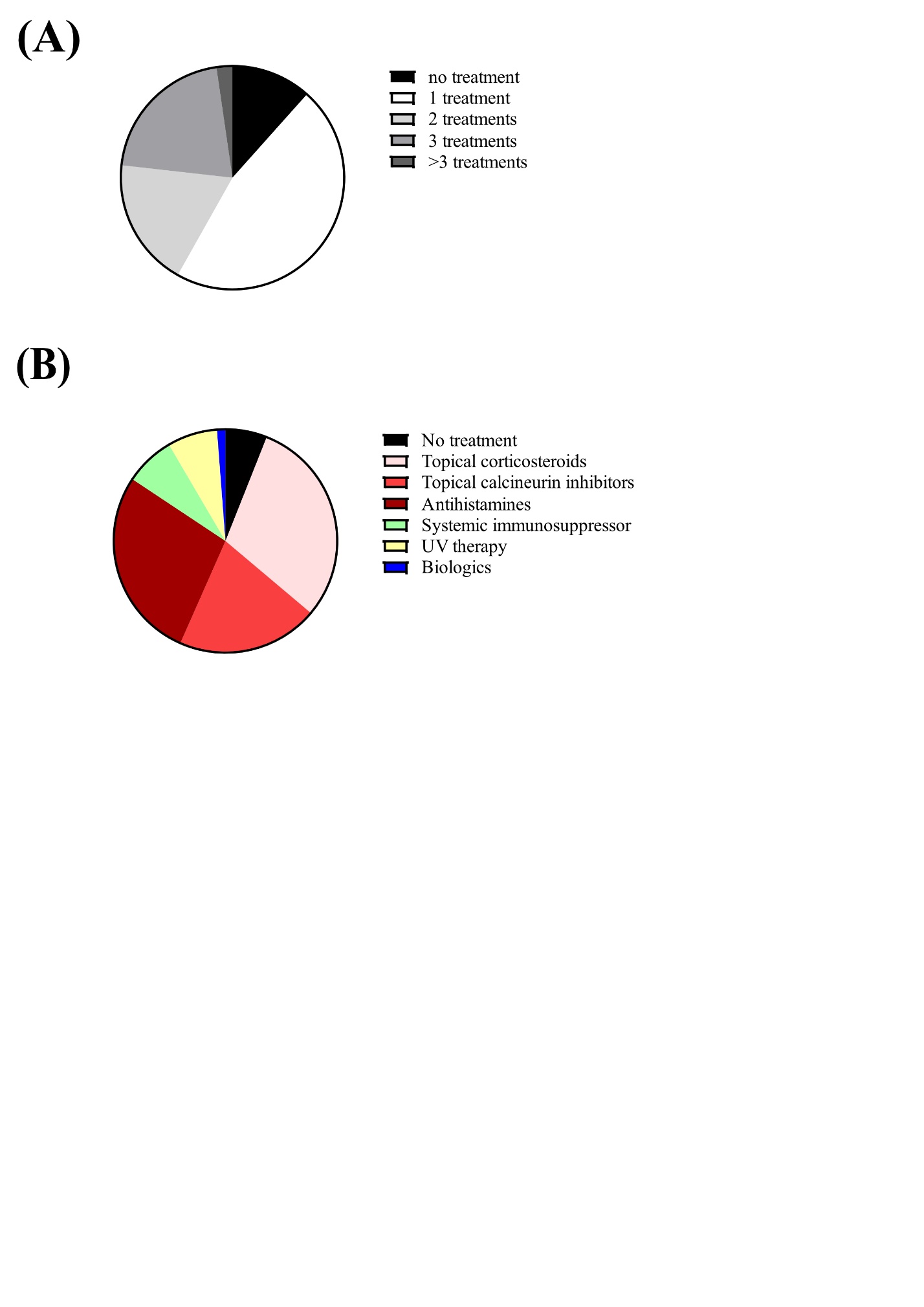
**

**Figure S1**
